# Changes in Physical Fitness and Body Composition Associated with Physical Exercise in Patients with Myasthenia Gravis: A Longitudinal Prospective Study

**DOI:** 10.3390/jcm10174031

**Published:** 2021-09-06

**Authors:** Che-Cheng Chang, Yen-Kung Chen, Hou-Chang Chiu, Jiann-Horng Yeh

**Affiliations:** 1 Department of Neurology, Fu Jen Catholic University Hospital, Fu Jen Catholic University, New Taipei City 24352, Taiwan; changcc75@gmail.com; 2 Ph.D. Program in Nutrition and Food Sciences, Human Ecology College, Fu Jen Catholic University, New Taipei City 24205, Taiwan; 3 Department of Nuclear Medicine and PET Center, Shin Kong Wu Ho-Su Memorial Hospital, Taipei 11101, Taiwan; M004149@ms.skh.org.tw; 4 School of Medicine, Fu Jen Catholic University, New Taipei City 24205, Taiwan; m001012.hc@gmail.com; 5 School of Medicine, Taipei Medical University, Taipei 11031, Taiwan; 6 Department of Neurology, Taipei Medical University, Shuang-Ho Hospital, New Taipei City 23561, Taiwan; 7 Department of Neurology, Shin Kong Wu Ho-Su Memorial Hospital, Taipei 11101, Taiwan; 8 Department of Neurology, Kaohsiung Medical University, Kaohsiung 80708, Taiwan

**Keywords:** body composition, myasthenia gravis, dual-energy X-ray absorptiometry, neuromuscular disease, physical exercises

## Abstract

There is a lack of guidelines for physical exercise in patients with myasthenia gravis (MG). A few pilot studies have shown that exercise can be safely applied to patients with MG. However, how physical exercise affects body composition, disease function, and disease severity remains unknown. In this prospective study, we enrolled 34 patients with MG with stable condition and evaluated the disease severity, physical fitness parameters, and body composition (measured using whole-body dual-energy X-ray absorptiometry (DXA)), before and after conducting a 24-week physical exercise regimen of aerobic and resistance strength training. The outcomes were measured by DXA, quantitative MG (QMG) score, quality of life score, handgrip strength and walking speed. During the training regimen, participants were free to decide how many exercise sessions per week and regularly reported their weekly exercise time. The physical exercise program was well tolerated by the participants, the parameters of the QMG score and handgrip strength improved, and participants’ body composition did not change significantly. The high exercise group experienced greater deterioration in muscle mass in the arms, but exhibited a greater improvement in forced vital capacity, walking speed, and symptom severity. The group with low QMG scores improved more in terms of physical fitness, including walking speed. These findings indicate that physical exercise is well tolerated by patients with MG, and is accompanied by improved muscular and physical functions. We propose that physical exercise is safe, effective, and appropriate for patients with well-regulated MG.

## 1. Introduction

Myasthenia gravis (MG) is an autoimmune disorder that can cause muscle weakness [1]. Fluctuating muscle weakness is the cardinal manifestation, which can take the form of ptosis, diplopia, dysphagia, dyspnea, or limb weakness. These vary over time and can lead to exercise intolerance. This exercise-induced fatigability can be observed clinically in patients with MG but has not been confirmed in controlled studies [2]. Current treatment for MG includes symptomatic treatment with anticholinergic medication along with immunosuppressants and thymectomy [3,4]. Currently, these treatment strategies are effective for improving muscle strength and survival rates. However, although these medications are well recognized to provide good symptom management, they have several side effects. Some patients experience poor symptom control even with multiple medications and occasionally require recurrent admission or ventilator support, leading to decreased quality of life. Hence patients may be unable to return to pre-morbid level of function.

Decreased physical activity can be a risk factor for developing chronic diseases, such as obesity and musculoskeletal disorders [5]. The benefits of physical exercise for chronic diseases are well documented. Previous evidence demonstrates at least 20–30% risk reductions for premature mortality and chronic disease among people who exercise according to international guidelines [6]. Resistance exercise training is an effective way to counteract muscle mass loss [7], and is effective for patients with MG. However, patients with MG are commonly instructed to avoid exercise due to the phenomenon of “overuse weakness” [2]. Further, some clinicians recommend restriction of physical exercise for patients with MG, given that overuse can overwhelm already weak muscles. There is a lack of clinical consensus for guidelines regarding the effect of exercise among patients with MG, and this issue is challenging for physicians and patients [8]. A meta-analysis revealed that strengthening and aerobic exercises are effective for patients with muscle disorders [9]. Because the patient’s capacity for exercise may be restricted by limb or respiratory muscle weakness [10], evaluation of the patient’s adaptability to physical exercise and its intensity are important. Multiple exercise programs of different intensities have been evaluated in several studies, but the most feasible exercise intensity for patients with MG remains uncertain. 

Body composition changes are a feature of the aging process including sarcopenia and obesity. Sarcopenia presents with progressive muscle wasting, decreased muscle strength, and poor physical fitness [11], and can also develop secondary to other etiologies, including some neuromuscular diseases [12]. Obesity is another condition that has been associated with various comorbidities and metabolic abnormalities [13]. MG disrupts the structure and function of the neuromuscular junction, leading to muscle weakness and changes in body composition. These changes include reduced muscle mass, increased adiposity, and an increased frequency of obesity [14]. Additionally, MG and sarcopenia have similar characteristics, which involve disruption of the neuromuscular junction and consequent functional changes in muscles [15]. Depending on the course of the disease and how it is managed, patients with MG may develop body composition changes such as sarcopenia and obesity, which have been associated with adverse outcomes and prognosis. Thus, understanding body composition changes as a result of exercise training is essential for assessing the safety and outcomes of physical exercise as a novel management strategy for patients with MG.

A literature review found several objective tools for evaluating the muscle functional changes due to exercise, including motor nerve conduction, neuromuscular ultrasound, and anthropometrics. The International Society for Clinical Densitometry recommends dual-energy X-ray absorptiometry (DXA) for accurately measuring body composition [16]. The advantages of DXA include speed, ease of use, and low radiation exposure [17]. DXA has been validated as a tool to precisely measure fat and muscle mass. The results obtained by DXA are also relatively consistent and not affected by human factors [17]. Using this accurate method of measurement of site-specific body composition may provide a better understanding of the role of exercise in MG. However, there is lack of study focus on the body composition changes in MG. Only one cross-sectional study using DXA demonstrates that the body composition changes in MG, including the increase body fat mass and body fat percentage [14].

To date, a few studies have focused on exercise training in patients with MG [18,19,20,21]; however, to the best of our knowledge, no studies have focused on the effects of exercise on body composition changes after exercise in patients with MG. In addition, understanding changes in body composition and disease course resulting from exercise training is essential for assessing the outcomes and safety of physical exercise, especially in patients with sarcopenia or obesity. Consequently, we aimed to clarify the effect of physical exercise on body composition (including muscle percentage and fat distribution), muscle strength, and physical fitness in patients with MG. 

## 2. Subjects and Methods

### 2.1. Study Design and Ethical Considerations

This prospective, unblinded study included patients with MG who were followed up at the Neurology Outpatient Clinic of the Shin Kong Wu Ho-Su Memorial Hospital, Taiwan, during 2018 and had undergone whole-body dual-energy X-ray absorptiometry (DXA). 

### 2.2. Participants

The inclusion criteria for patients with MG were (1) Myasthenia Gravis Foundation of America (MGFA) Class II and III, and (2) no medication adjustment in the last 6 months. The exclusion criteria were: (1) unstable MG symptoms, (2) history of intensive immunotherapy, including immunoglobulins or plasmapheresis, 6 months before enrollment. Patients were eligible if they were diagnosed with MG based on MGFA criteria [22]. Briefly, the diagnosis of MG was based on fluctuating muscle weakness with fatigability, decreased symptom severity after use of acetylcholinesterase inhibitors, decremental changes in repetitive nerve stimuli on repetitive nerve stimulation test, or presence of autoantibodies against the acetylcholine receptor (AChR) [22]. Among individuals who met the inclusion criteria, 35 participants agreed to participate. This study complied with the principles of the Declaration of Helsinki and was approved by the ethical committee of the Shin Kong Wu Ho-Su Memorial Hospital (No. 20170914R). All participants in the MG group provided written informed consent before enrollment in the study.

A previous study had enrolled 7 patients with MG who completed a pre- and post-intervention, which was 16 sessions of balance strategy training [23]. Of these, five had complete QMG score data. The mean and standard deviation was 10 ± 4.43 at pre-intervention and 6.6 ± 3.21 at post-intervention, resulting in a standardized mean difference (SMD) of 0.86. Given the effect size of 0.86, we calculated that a minimum sample size of 27 was required in the present study to achieve a Type I error of 5% and a power of 99%.

### 2.3. Physical Exercise Regimen

We developed individually tailored 24-week physical therapy plans based on general physical exercise recommendations for healthy adults [24]. Each exercise session lasted 30 min. Every session consisted of aerobic resistance training in a physiotherapy setting at Shin Kong Wu Ho-Su Memorial Hospital. It has been demonstrated that aerobic resistance training can induce improvements in both aerobic fitness and anaerobic capacity with very little time commitment [25]. Participants were instructed to perform the exercise cycle once a month while supervised by a researcher. They were then instructed to perform the same session at home, time and record the sessions completed, and report the number of sessions (1 session = 30 min) and the number of days spent for exercise in a week to the researchers. 

The physical exercise program selected in this study was designed by the researchers. Training started with a 5-min warmup followed by seven cycling intervals of 3 min each and ending with a 5-min cool-down. Aerobic resistance training included squat, sit-to-stand, arms-out stretch, squat jumps, sprint in place, and exercises using one’s body weight. If the participant was able to complete the exercises easily, the intensity was gradually increased by increasing the number of repetitions and altering the speed. The active training program was followed by a set of stretching exercises. None of the exercises were used to measure the effect of the intervention. During the training regimen, participants were free to decide how many exercise sessions per week they would perform and regularly reported their weekly exercise time. 

### 2.4. Outcome Measures

All clinical and muscle function evaluations were performed before and after the 24-week physical exercise training period. 

### 2.5. Clinical Measures

Information on participants’ medical history, including the average daily dose of corticosteroids and all other medications taken, was collected at the time of evaluation. Clinical status and MG severity were determined according to the recommendations of the MGFA [24]. Trained researchers assessed the quantitative MG (QMG) scores, including handgrip test and forced vital capacity (FVC), and MG quality of life (MG-QOL) scores, according to previously established methodology [26,27]. Body mass index (BMI) was calculated as body weight in kilograms divided by height in meters squared (kg/m^2^). Daily doses of prednisone and other immunosuppressants were extracted from the medical records. The QMG scores is a scoring system that quantifies MG disease severity. Each item is quantitatively assessed and scored from 0 to 3, providing a total QMG scores ranging from 0 to 39. The QMG scores is composed several function assessments including ocular, facial, bulbar, limb muscle, axial muscle, and respiratory muscle function.

### 2.6. Definition of Sarcopenia, Obesity, and Sarcopenic Obesity

Based on the DXA data, sarcopenia was defined as an ASMI < 7.0 kg/m^2^ for men or 5.4 kg/m^2^ for women, according to the criteria for Asian populations [28]. Obesity was defined if one of these four conditions were met: high A/G ratio (>0.80 in men, >0.62 in women), high android fat mass (>2.16 kg for men, >1.95kg for women), high body fat percentage (>31.8% for men, >38.8% for women), or BMI > 25 kg/m^2^, according to previous cohort studies in Asian populations [29]. Sarcopenic obesity was assigned if both criteria for obesity and sarcopenia were met.

### 2.7. Body Composition Assessment

Body composition was assessed using DXA by certified radiological technologists. Briefly, images were obtained in the supine position and analyzed according to the manufacturer’s specifications. We evaluated the following parameters: appendicular (arms and legs) fat mass (kg); appendicular lean muscle mass (kg); arm, leg, appendicular, android, gynoid, and whole-body adiposity (%); arm, leg, appendicular, android, gynoid, and whole-body lean muscle mass percentage (%); and appendicular skeletal muscle mass (ASM, kg). The ASM index (ASMI) was calculated by dividing the ASM (fat-free mass in the arms and legs; kg) by the height squared (m^2^). The android-to-gynoid (A/G) ratio was calculated as the ratio of android adiposity to gynoid adiposity.

### 2.8. Physical Fitness Measures

Gait speed (6-m timed walk (6MTW)), handgrip strength test, and FVC were assessed by researchers before and after the 24-week training period. The 6MTW was performed in the physiotherapy department twice on both occasions to avoid introducing a learning effect, and the average speed was used for analysis. The participant was instructed to walk at their preferred speed. The handgrip strength test was repeated twice and the average taken for analysis.

### 2.9. Statistical Analyses

The changes in body composition before and after resistance training were tested using a paired sample *t*-test. The patients with MG were further divided into several dichotomized subgroups according to the median time spent on exercise per week. We divided the participants into a high exercise group (above the median exercise time) and a low exercise group (below the median exercise time), as well as according to the presence or absence of sarcopenia, the presence or absence of obesity, and low or high QMG score (≤10 vs. >11). The changes in body composition before and after resistance training in each subgroup were also tested using a paired sample *t*-test. The effect size (standardized mean difference (SMD)) before and after resistance training was also reported, in which an absolute SMD value >0.2, >0.5 or >0.8 was considered a small, medium, or large difference, respectively. The subgroup difference in the change of body composition before and after resistance training was tested using a generalized estimating equation (GEE) that included the intercept, main effects of time and subgroup, and a two-way interaction term of ‘time by subgroup’. The change in body composition before and after resistance training between subgroups was considered to be different when the interaction effect was significant. 

All tests were 2-tailed, and statistical significance was set at *p* < 0.05. No adjustment for multiple testing (multiplicity) was made in this study. Data analyses were conducted using SPSS 25 (IBM SPSS Inc., Chicago, IL, USA).

## 3. Results

### 3.1. Clinical Features of the 35 MG Patients

Thirty-five patients with MG, including 13 men and 22 women, were included in this study. The mean age was 56.1 years (standard deviation (SD) = 8.6 years). All patients were positive for AChR autoantibodies. Bulbar involvement was found in 28.6% (*n* = 10) and 25.7% (*n* = 9) had had previous myasthenic crises requiring ICU hospitalization. The average disease duration was 12.3 years (SD = 10.6 years). Forty percent (*n* = 14) of patients with MG were obese, approximately one-fifth (*n* = 8) were sarcopenic, and only one patient had sarcopenic obesity. Fourteen patients with MG (40%) had been prescribed steroids for 6 months, with an average daily dose of 5.3 mg (SD = 5.7 mg). Ten patients with MG (28.6%) received immunosuppressant treatment. The sample comprised 21 (60%) MGFA Type II patient with and 14 (40%) MGFA Type III patients with MG (Table 1).

### 3.2. Changes in Body Composition before and after Resistance Training

**Figure 1:**
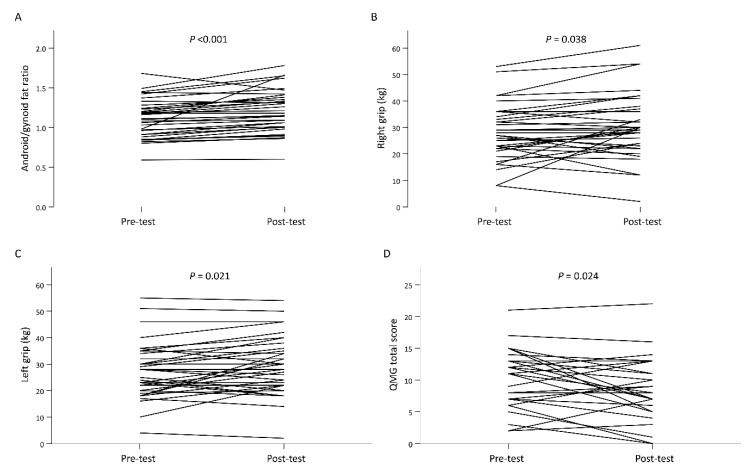
The individual data of android/gynoid fat ratio (**A**), right handgrip strength (**B**), left handgrip strength (**C**) and QMG total score (**D**) from the baseline to the end of the 24-week training period. QMG, quantitative myasthenia gravis.

DXA-derived body composition measures for patients with MG before and after resistance training programs are shown in Table 2. Finally, 34 patients with MG, including 13 men and 21 women, were enrolled in exercise training regimen and one patient gave up participating in our study. Android/gynoid fat ratios significantly increased (1.11 vs. 1.20; mean difference (MD) 0.1, 95% confidence interval (CI) 0.05 to 0.14) with a small to medium effect size (SMD = 0.38). In addition, both right and left grip significantly improved from the baseline to the end of the 24-week training period, with a small effect size (SMD = 0.22). Noticeably, the QMG total score significantly improved after the intervention (10.47% vs. 9.0: MD, −1.47; 95% CI, −2.73 to 0.21) with a small effect size (SMD = −0.29). Figure 1 illustrates the individual data of the significant items from the baseline to the end of the 24-week training period. Additionally, there were no significant changes in the remaining body composition measures after resistance training (Table 2).

### 3.3. Subgroup Analysis According to the Time Spent Exercising per Week

**Figure 2:**
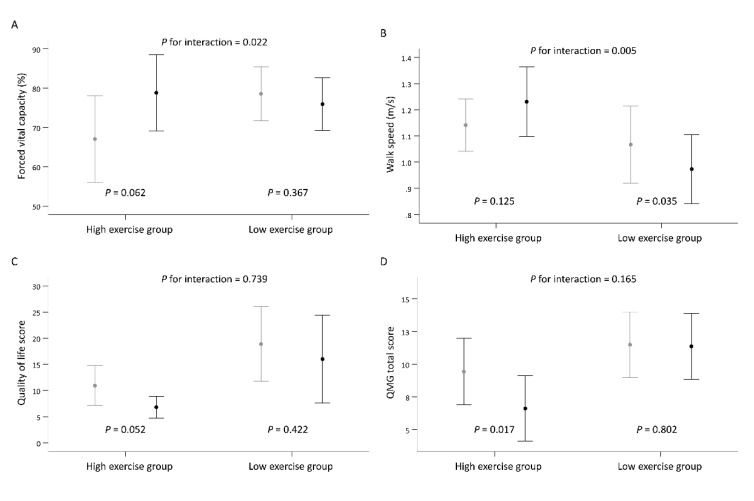
The grouped data of forced vital capacity, walking speed, QMG and QOL scores from the baseline to the end of the 24-week training period stratified by median time spent exercising per week. There is significance difference between groups in force vital capacity (**A**) and walking speed (**B**), the high exercise group also showed significance improvement in QOL score (**C**) and QMG score (**D**) after exercise training. QMG, quantitative myasthenia gravis; QOL, quality of life.

The median time spent exercising in our cohort was 56.3 min per week, and the median number sessions was 2.9 (interquartile range (IQR), 1.1–4.5 sessions). We further divided the patients into a high exercise (above median exercise time) and a low exercise group (below median exercise time). The results with the GEE model demonstrated that the high exercise group experienced greater deterioration in the muscle mass of the arms (MD, −0.35 kg; 95% CI −0.68 to −0.03 kg) after intervention than did the low exercise group. However, the high exercise group exhibited a greater improvement in forced vital capacity (MD, 14.39%; 95% CI, 2.05 to 26.72%) and walking speed (MD, 0.18 m/s, 95% CI, 0.05 to 0.31 m/s) between baseline and the 24th week than did the low exercise group (Table 3). Additionally, the QMG and QOL scores improved significantly in the high exercise group. Figure 2 illustrates the grouped data of forced vital capacity, walking speed, QMG and QOL scores from the baseline to the end of the 24-week training period stratified by median time spent exercising per week.

### 3.4. Subgroup Analysis by the Presence or Absence of Sarcopenia and Obesity

Analysis of the subgroups according to sarcopenia indicated that the effect of the resistance training program on all body composition measures was consistent among patients with and without sarcopenia; however, QMG scores decreased significantly after resistance training in the non-sarcopenia group (Table 4). Similarly, the effect of the resistance training program on all body composition measures did not vary between patients with and without obesity (Table 5).

### 3.5. Subgroup Analysis of the QMG Total Score

A further subgroup analysis was performed using different levels of QMG scores. This revealed that patients with higher QMG scores (>11) demonstrated a greater increase in BMI values compared to patients with lower scores (≤10) after intervention (MD, 0.68 kg/m^2^, 95% CI, 0.01 to 1.35 kg/m^2^). Alternatively, the group with low QMG scores displayed greater improvement in walk speed (MD, -0.18 m/s, 95% CI, −0.32 to −0.05 m/s) after intervention than did the group with higher QMG scores (Table 6).

## 4. Discussion

To the best of our knowledge, this is the first study to use whole-body composition determination by DXA as a tool for evaluating body composition changes after physical exercise in patients with MG. We evaluated the physical fitness and body composition changes in patients with MG after resistance training with different intensities of exercise and disease severities. We observed that physical exercise is feasible for most patients with MG and our training program was well tolerated. The DXA indicated that the prevalence of sarcopenia was 8%, obesity 40%, and sarcopenic obesity 1% among patients with MG. Although A/G ratios increased after the 24-week physical exercise program, the handgrip strength and QMG scores significantly improved after the intervention. According to the subgroup analyses, the high exercise group experienced not only improvement in physical fitness, such as vital capacity and walking speed, but also decreased QMG and QOL scores. The improvement in QMG scores was more pronounced among non-sarcopenic patients with MG, even though there were no differences in body composition changes between the subgroups. The lower the QMG score, the lower the appendicular, gynoid, and whole-body muscle mass percentages. Although the change was not significant, the QOL score decreased more prominently among the low QMGS group. 

The QMG scores and handgrip strength improved significantly after the 24-week physical exercise program (QMG score improvement from 10.47 to 9.0), and the high exercise group demonstrated a greater decrease in QMG and QOL scores than did the lower exercise group, which indicates a clinically significant improvement in MG symptoms post-intervention. No participants reported negative effects due to the training program. Our results are compatible with findings from previous studies in the MG-QOL after exercise training. An observational study of 14 patients with MG reported a slight decrease in QMG scores after physical exercise training combined with improvement in QOL [20]. Because QMG, QOL and handgrip strength (part of QMG scores assessment) can represent MG severity and functional impairment, our results indicate continual improvement in function, strength, and endurance in daily function. Exercise may provide benefits through several physiological effects, including accelerated lactate metabolism, increased muscle strength, and increased number of mitochondria [30]. Neuromuscular transmission can also be improved after physical exercise, which can increase the resistance of muscles to fatigue and improve endurance [31].

We observed improvement in FVC. Respiratory insufficiency, which is caused by diaphragm weakness, can be life-threatening for patients with MG, who often experience reduced vital capacity [1]. A decrease in vital capacity and forced expiratory volume in 1 s (FEV1) was observed even in patients with well-controlled myasthenia syndrome [10]. Respiratory distress can increase fatigue and lead to exercise intolerance. Previous studies have shown that changes in physical activity habits are associated with an improvement in FEV1 and FVC in patients with restrictive pulmonary disease [32]. A systematic review demonstrated that breathing exercises had potential benefits for patients with MG [9] and expiratory muscle strength could be an important predictor of the walk distance test [33]. Other parameters of physical fitness, including walking speed, also improved in the high exercise group. One case series study revealed an improvement in walking distance after exercise training in patients with MG [23]. A prospective pilot study by Westerberg et al. also indicated improvement in leg muscle strength and all functional outcomes as a result of physical exercise [20]; this result was also observed among healthy individuals [34]. The increase in leg muscle strength may lead to an improvement in walking speed and distance. Our participants also demonstrated improvement in walking speed. These results suggest an improvement in physical performance, which results in improved community mobility and participation. 

The improvement of all functional outcomes observed in this study, including handgrip strength and walking speed, is consistent with previous studies of patients with MG and healthy individuals [18,19,35,36]. However, we also observed deterioration of the muscle mass of the arms in the high exercise group. Previous research indicates that physical training can improve muscle force, especially in the lower limbs, more so than in the upper limbs [35]. Westerberg et al. demonstrated similar results in that exercise had beneficial effects on muscle outcomes, especially in leg muscles, without improvement of upper limb function in patients with MG [20]. Other studies have shown that arm muscle strength improves after intervention [19]. A larger amount of leg muscle training in our regimen could be one explanation for the difference in observations, and some studies indicate that arm muscle requires a longer duration of training intervention to reach similar effects observed in the legs because the legs are naturally active on a daily basis [20]. Hence, we can speculate that a higher degree of arm inactivity at baseline would require a longer duration of training intervention to achieve similar improvement compared to the leg muscles. Furthermore, sex differences have been previously observed in that healthy females display higher compound muscle action potential amplitude increases in the quadriceps after training [34]. Our participants included more female patients with MG than males, which could possibly contribute to the result. 

The decrease in arm muscle mass we observed in the high-intensity exercise group can be accounted for in two ways. First is the muscle strength fatigue effect. A literature review revealed that after an eight-week study, fatigue in patients with MG increased after exercise training [18]. Although some fatigue is short-lived, the exercise method may worsen the symptoms of some patients with MG. Decreased muscle mass and fatigue are compatible with the hallmark symptoms of MG, and because the severity of symptoms of MG varies greatly [1], some changes in body composition may be caused by random fluctuations in symptoms, rather than the intervention. Second, muscle hypertrophy in the upper arms after heavy resistance training has been reported previously [37]. Wilmore et al. also demonstrated that high resistance training results in increased diameter in arm muscles more than in leg muscles, which is different from our results [38]. The difference may be due to our training regimen, in which the arm training may not be strong enough to cause an increase in muscle mass. Thus, further development of our regimen should focus on the arm training program. These factors may limit the degree of improvement observed in individual participants and may suggest a reason the results are not clinically significant.

Based on studies of patients with other autoimmune diseases, regular exercise is a cornerstone of care in addition to pharmacological treatment [39]. Lower physical activity levels are observed among these patients compared with the healthy population, and lifestyle modifications play a critical role in autoimmune function even under well-controlled by medical treatment [39]. General fatigue and decreased cardiovascular fitness are common among patients with neuromuscular diseases [2]. Exercise can enhance capacity, improve muscle function, and reduce disability in inflammatory neuromuscular diseases [40,41]. The mechanism of physical exercise can enhance physical function, including the following: (1) increase T regulatory cells, (2) change the Th1/Th2 balance, and (3) release of anti-inflammatory cytokines, including IL-6 [31]. Physical exercise could reduce adipose tissue and increase muscle mass, and can also increase aerobic capacity [42]. 

Understanding changes in body composition after physical exercise can assist with the development of a novel approach for health promotion and physical management of MG. Our analyses using DXA to explore the effect of physical exercise among patients with MG included 34 participants with MG, which is a larger sample than previous studies [18,19,20]. The basic symptoms of muscle fatigue among patients with MG often make physical therapists uncertain whether patients can safely comply with exercise and resistance training. The health risks associated with this fatigue, as well as the possible adverse health effects of drugs and disease-related diseases, should be carefully considered by clinicians and physical therapists. It is important to evaluate patients’ tolerance for and the effectiveness of different training strategies for the treatment of MG. Our findings indicated that MG patients tolerated exercise well, without a decrease in symptom severity and quality of life, which is consistent with previous studies on exercise in MG patients [43]. Nevertheless, this study supports that among patients with MG, long duration exercise training scores do not worsen MG symptoms or cause significant changes in whole-body muscle mass. Our physical exercise regimen also improved the quality of life and ameliorated the symptoms of MG. The findings of improved outcomes indicate that disease severity was well controlled, and the disease mechanism of MG does not interfere with the expected improvement in muscle parameters after training. In turn, this also indicates that the exercise plan applied in this study can be recommended to patients who desire to maintain exercise or physical activity, while living with MG. 

Our study had the following limitations. First, the sample size was low, and the results may not be representative of all patients with MG. Furthermore, statistical control of baseline factors between subgroups was not feasible due to the restricted sample size. Therefore, the interpretation of the conclusions from the subgroup analysis should be conservative and future larger-scale studies are warranted. Second, interventional studies such as this tend to waste a large amount of time that could have caused some variation in the patients. Third, some of the exercises and tests depended on individual motivation, which could limit the extent of improvement of muscle mass and muscle strength we observed. Forth, we did not collect information on the daily physical activity, nutritional status, and dietary habits of the study participants. Fifth, our intervention time could be considered too short to allow assessment of the long-term effects of physical training on patients with MG. Finally, because the amount of exercise taken per week was determined by the patient, the patients were not divided into exercise subgroups at study start, which prevents control of the baseline characteristics of the high-exercise and low-exercise groups. Future research should explore the detailed physical and pathological changes in the neuromuscular junction in relation to exercise. 

In summary, this longitudinal prospective study was the first to use DXA as a tool for the measurement of outcomes in patients with MG after a physical exercise program, and the results demonstrate that physical exercise can be safe and beneficial for functional outcomes among patients with MG. Additionally, we did not detect any clinical deterioration among our participants, and the QMG score, which represents MG severity, improved in the high exercise group. Given the lack of an established and effective exercise plan for patients with MG, it is necessary to develop an exercise plan to enhance the exercise function of these patients, and whole-body DXA could be a potential tool for monitoring the outcomes of patients with MG after exercise training. 

## Figures and Tables

**Table 1:** Clinical features of the 35 patients with myasthenia gravis.

Variable	*n* (%) or Mean ± Standard Deviation
Male sex	13 (37.1)
Age (year)	56.1 ± 8.6
Age group	
40–49 yrs.	10 (28.6)
50–59 yrs.	10 (28.6)
60–70 yrs.	15 (42.9)
Disease duration (year)	12.3 ± 10.6
Obesity	14 (40.0)
Sarcopenia	8 (22.9)
Sarcopenic obesity	1 (2.9)
Use of corticosteroids in the last six months	14 (40.0)
Corticosteroid daily dose in recent six months (mg)	5.3 ± 5.7
Immunosuppressant used	10 (28.6)
MGFA type	
Type II	21 (60)
Type III	14 (40)

MGFA, Myasthenia Gravis Foundation of America.

**Table 2:** Body composition of patients with MG before and after resistance training (*n* = 34).

Variable	Pre-Test (*n* = 34)	Post-Test (*n* = 34)	Mean Difference (95% CI)	*p*
Body mass index (kg/m^2^)	24.80 ± 4.62	24.86 ± 4.50	0.06 (−0.29, 0.41)	0.733
Fat mass (kg)				
Arms	2.05 ± 0.92	2.07 ± 0.88	0.01 (−0.12, 0.15)	0.819
Legs	6.68 ± 2.71	6.85 ± 3.29	0.17 (−0.52, 0.86)	0.623
Appendicular	8.73 ± 3.42	8.91 ± 3.95	0.18 (−0.53, 0.90)	0.605
Muscle mass(kg)				
Arms	4.29 ± 1.49	4.28 ± 1.30	−0.01 (−0.19, 0.17)	0.925
Legs	13.70 ± 3.11	13.56 ± 3.31	−0.13 (−0.42, 0.16)	0.357
Appendicular	17.99 ± 4.43	17.85 ± 4.53	−0.14 (−0.54, 0.26)	0.478
Fat adiposity (%)				
Android	43.26 ± 9.45	44.84 ± 9.98	1.58 (−0.20, 3.35)	0.079
Gynoid	39.78 ± 7.59	38.76 ± 9.25	−1.02 (−2.63, 0.60)	0.209
Muscle (%)				
Arms	67.91 ± 10.79	67.85 ± 10.72	−0.06 (−0.98, 0.85)	0.888
Legs	67.80 ± 8.50	67.28 ± 10.38	−0.52 (−2.42, 1.37)	0.579
Appendicular	67.77 ± 8.63	67.29 ± 10.15	−0.48 (−2.13, 1.16)	0.556
Android	56.74 ± 9.45	55.16 ± 9.98	−1.58 (−3.35, 0.20)	0.079
Gynoid	60.22 ± 7.59	61.24 ± 9.25	1.02 (−0.60, 2.63)	0.209
Whole body	65.51 ± 9.10	65.72 ± 8.09	0.21 (−1.39, 1.82)	0.787
Android/gynoid fat ratio	1.11 ± 0.24	1.20 ± 0.27	0.10 (0.05, 0.14)	<0.001 *
Body fat percentage (%)	34.10 ± 8.47	34.33 ± 8.06	0.23 (−0.60, 1.06)	0.576
ASMI	6.66 ± 1.21	6.59 ± 1.20	−0.06 (−0.20, 0.07)	0.340
Forced vital capacity (%)	72.64 ± 18.48	77.42 ± 16.28	4.79 (−2.36, 11.93)	0.182
Walk speed (m/s)	1.10 ± 0.25	1.10 ± 0.28	−0.01 (−0.08, 0.07)	0.852
Right grip (kg)	27.82 ± 10.58	30.35 ± 12.25	2.53 (0.15, 4.91)	0.038 *
Left grip (kg)	27.21 ± 10.79	29.56 ± 11.02	2.35 (0.37, 4.34)	0.021 *
Quality of life score (*n* = 32)	14.91 ± 11.29	11.41 ± 12.24	−3.50 (−7.51, 0.51)	0.085
QMG total score (*n* = 32)	10.47 ± 4.78	9.00 ± 5.22	−1.47 (−2.73, −0.21)	0.024 *

MG, myasthenia gravis; CI, confidence interval; QMGS, quantitative myasthenia gravis scores. * Significantly different before and after training in the MG groups.

**Table 3:** Body composition of patients with MG before and after resistance training according to the time spent exercising per week (by median).

Variable	High Exercise Group (≥56.3 Min/Week)			Low Exercise Group (<56.3 Min/Week)			Mean Difference (95% CI)	*p* for Interaction
	Pre-Test (*n* = 17)	Post-Test (*n* = 17)	*p*	Pre-Test (*n* = 17)	Post-Test (*n* = 17)	*p*		
Body mass index (kg/m^2^)	23.85 ± 4.46	23.91 ± 4.40	0.862	25.74 ± 4.71	25.81 ± 4.52	0.718	−0.01 (−0.68, 0.66)	0.972
Fat mass (kg)								
Arms	1.81 ± 0.91	1.78 ± 0.85	0.685	2.30 ± 0.88	2.35 ± 0.83	0.619	−0.08 (−0.33, 0.16)	0.505
Legs	6.30 ± 2.54	6.78 ± 3.73	0.477	7.06 ± 2.89	6.92 ± 2.90	0.366	0.63 (−0.67, 1.92)	0.342
Appendicular	8.11 ± 3.35	8.56 ± 4.38	0.507	9.36 ± 3.46	9.27 ± 3.57	0.701	0.54 (−0.80, 1.88)	0.429
Muscle mass (kg)								
Arms	4.70 ± 1.52	4.52 ± 1.44	0.074	3.88 ± 1.37	4.05 ± 1.15	0.251	−0.35 (−0.68, −0.03)	0.033 *
Legs	14.29 ± 3.26	14.07 ± 3.69	0.399	13.10 ± 2.92	13.06 ± 2.90	0.743	−0.18 (−0.72, 0.36)	0.511
Appendicular	18.99 ± 4.73	18.58 ± 5.06	0.204	16.98 ± 4.00	17.11 ± 3.94	0.589	−0.54 (−1.27, 0.20)	0.152
Fat adiposity (%)								
Android	38.84 ± 9.11	41.33 ± 11.01	0.149	47.67 ± 7.74	48.34 ± 7.64	0.258	1.83 (−1.48, 5.14)	0.278
Gynoid	37.40 ± 7.17	36.99 ± 10.37	0.783	42.16 ± 7.45	40.54 ± 7.88	0.013	1.20 (−1.84, 4.24)	0.439
Muscle (%)								
Arms	73.23 ± 9.60	72.35 ± 10.31	0.215	62.59 ± 9.37	63.34 ± 9.35	0.187	−1.62 (−3.28, 0.03)	0.054
Legs	69.94 ± 8.31	68.48 ± 12.24	0.430	65.65 ± 8.39	66.07 ± 8.32	0.363	−1.89 (−5.43, 1.66)	0.297
Appendicular	70.65 ± 8.38	69.25 ± 11.72	0.382	64.88 ± 8.10	65.32 ± 8.18	0.298	−1.84 (−4.90, 1.22)	0.239
Android	61.16 ± 9.11	58.67 ± 11.01	0.149	52.33 ± 7.74	51.66 ± 7.64	0.258	−1.83 (−5.14, 1.48)	0.278
Gynoid	62.60 ± 7.17	63.01 ± 10.37	0.783	57.84 ± 7.45	59.46 ± 7.88	0.013	−1.20 (−4.24, 1.84)	0.439
Whole body	69.21 ± 8.00	69.04 ± 7.37	0.776	61.80 ± 8.81	62.40 ± 7.56	0.689	−0.77 (−3.80, 2.25)	0.616
Android/gynoid fat ratio	1.06 ± 0.27	1.14 ± 0.27	0.003	1.15 ± 0.21	1.26 ± 0.26	0.012	−0.03 (−0.12, 0.05)	0.457
Body fat percentage (%)	31.08 ± 7.68	31.06 ± 7.37	0.974	37.12 ± 8.35	37.59 ± 7.55	0.452	−0.49 (−2.06, 1.07)	0.535
ASMI	6.88 ± 1.25	6.76 ± 1.29	0.161	6.43 ± 1.16	6.42 ± 1.12	0.967	−0.12 (−0.38, 0.13)	0.354
Forced vital capacity (%)	67.06 ± 21.47	78.82 ± 18.83	0.062	78.56 ± 12.82	75.94 ± 13.52	0.367	14.39 (2.05, 26.72)	0.022 *
Walk speed (m/s)	1.14 ± 0.20	1.23 ± 0.25	0.125	1.07 ± 0.29	0.97 ± 0.26	0.035	0.18 (0.05, 0.31)	0.005 *
Right grip (kg)	29.65 ± 11.30	31.53 ± 13.82	0.247	26.00 ± 9.80	29.18 ± 10.77	0.093	−1.29 (−5.80, 3.21)	0.573
Left grip (kg)	28.94 ± 11.87	29.94 ± 12.46	0.552	25.47 ± 9.63	29.18 ± 9.74	0.002	−2.71 (−6.36, 0.95)	0.146
Quality of life score (*n* = 32)	10.94 ± 7.16	6.81 ± 3.89	0.052	18.88 ± 13.36	16.00 ± 15.80	0.422	−1.24 (−8.51, 6.04)	0.739
QMG total score (*n* = 32)	9.44 ± 4.77	6.63 ± 4.67	0.017	11.50 ± 4.70	11.38 ± 4.73	0.802	−1.76 (−4.26, 0.73)	0.165

MG, myasthenia gravis; CI, confidence interval; QMGS, quantitative myasthenia gravis scores. * Significantly different in the high exercise group compared to the low exercise group.

**Table 4:** Body composition of patients with MG before and after resistance training according to presence or absence of sarcopenia.

Variable	Sarcopenia			Non-Sarcopenia			Mean Difference (95% CI)	*p* for Interaction
	Pre-Test (*n* = 8)	Post-Test (*n* = 8)	*p*	Pre-Test (*n* = 26)	Post-Test (*n* = 26)	*p*		
Body mass index (kg/m^2^)	21.71 ± 2.32	21.50 ± 2.51	0.617	25.75 ± 4.76	25.89 ± 4.49	0.465	−0.35 (−1.18, 0.47)	0.401
Fat mass (kg)								
Arms	1.58 ± 0.53	1.66 ± 0.57	0.197	2.20 ± 0.97	2.19 ± 0.92	0.933	0.09 (−0.10, 0.29)	0.345
Legs	4.99 ± 1.32	5.15 ± 1.29	0.503	7.20 ± 2.83	7.37 ± 3.55	0.700	−0.01 (−0.95, 0.92)	0.975
Appendicular	6.57 ± 1.38	6.81 ± 1.42	0.394	9.40 ± 3.59	9.56 ± 4.26	0.720	0.08 (−0.92, 1.08)	0.879
Muscle mass(kg)								
Arms	3.37 ± 0.57	3.50 ± 0.61	0.088	4.58 ± 1.57	4.52 ± 1.37	0.652	0.19 (−0.07, 0.44)	0.149
Legs	11.39 ± 1.53	11.46 ± 1.44	0.631	14.40 ± 3.14	14.21 ± 3.47	0.291	0.26 (−0.16, 0.69)	0.228
Appendicular	14.76 ± 2.01	14.96 ± 2.01	0.252	18.98 ± 4.52	18.73 ± 4.74	0.332	0.45 (−0.11, 1.01)	0.118
Fat adiposity (%)								
Android	40.29 ± 11.29	41.87 ± 10.21	0.305	44.17 ± 8.86	45.75 ± 9.94	0.151	0.003 (−3.33, 3.33)	0.999
Gynoid	38.69 ± 7.24	37.36 ± 7.08	0.268	40.12 ± 7.81	39.20 ± 9.90	0.361	−0.41 (−3.18, 2.37)	0.774
Muscle (%)								
Arms	68.15 ± 9.65	67.88 ± 9.87	0.783	67.84 ± 11.29	67.84 ± 11.15	0.998	−0.28 (−2.30, 1.75)	0.789
Legs	69.56 ± 7.71	69.07 ± 7.37	0.621	67.26 ± 8.80	66.72 ± 11.21	0.659	−0.04 (−2.84, 2.92)	0.979
Appendicular	69.05 ± 7.11	68.59 ± 7.00	0.646	67.37 ± 9.14	66.88 ± 11.02	0.638	0.03 (−2.60, 2.66)	0.982
Android	59.71 ± 11.29	58.13 ± 10.21	0.305	55.83 ± 8.86	54.25 ± 9.94	0.151	−0.003 (−3.33, 3.33)	0.999
Gynoid	61.31 ± 7.24	62.64 ± 7.08	0.268	59.88 ± 7.81	60.80 ± 9.90	0.361	0.41 (−2.37, 3.18)	0.774
Whole body	66.14 ± 11.04	67.45 ± 7.99	0.689	65.31 ± 8.67	65.19 ± 8.19	0.786	1.43 (−4.39, 7.25)	0.629
Android/gynoid fat ratio	1.06 ± 0.26	1.14 ± 0.30	0.041	1.12 ± 0.24	1.22 ± 0.26	0.002	−0.01 (−0.10, 0.07)	0.765
Body fat percentage (%)	32.23 ± 8.12	32.64 ± 7.96	0.689	34.67 ± 8.65	34.85 ± 8.17	0.702	0.24 (−1.77, 2.25)	0.815
ASMI	5.63 ± 0.67	5.74 ± 0.58	0.241	6.97 ± 1.17	6.85 ± 1.23	0.160	0.23 (0.01, 0.45)	0.044
Forced vital capacity (%)	76.29 ± 12.83	82.14 ± 9.77	0.160	71.65 ± 19.82	76.15 ± 17.57	0.313	0.86 (−9.86, 11.57)	0.876
Walk speed (m/s)	1.06 ± 0.25	1.15 ± 0.42	0.322	1.12 ± 0.25	1.08 ± 0.23	0.343	0.13 (−0.04, 0.30)	0.146
Right grip (kg)	26.75 ± 6.80	29.25 ± 6.02	0.140	28.15 ± 11.59	30.69 ± 13.70	0.098	−0.04 (−3.99, 3.91)	0.985
Left grip (kg)	26.00 ± 6.93	27.63 ± 5.71	0.368	27.58 ± 11.82	30.15 ± 12.23	0.038	−0.95 (−4.79, 2.88)	0.627
Quality of life score (*n* = 32)	11.29 ± 7.30	11.43 ± 11.13	0.957	15.92 ± 12.10	11.40 ± 12.75	0.071	3.47 (−3.18, 10.12)	0.306
QMG total score (*n* = 32)	7.57 ± 4.20	8.29 ± 4.99	0.593	11.28 ± 4.69	9.20 ± 5.36	0.005	1.78 (−0.94, 4.50)	0.201

MG, myasthenia gravis; CI, confidence interval; QMGS, quantitative myasthenia gravis scores.

**Table 5:** Body composition of patients with MG before and after resistance training according to obesity and non-obesity.

Variable	Obesity			Non-Obesity			Mean Difference (95% CI)	*p* for Interaction
	Pre-Test (*n* = 14)	Post-Test (*n* = 14)	*p*	Pre-Test (*n* = 20)	Post-Test (*n* = 20)	*p*		
Body mass index (kg/m^2^)	29.11 ± 3.67	29.12 ± 3.29	0.977	21.77 ± 2.13	21.87 ± 2.21	0.650	−0.09 (−0.79, 0.62)	0.812
Fat mass (kg)								
Arms	2.74 ± 0.77	2.71 ± 0.72	0.838	1.57 ± 0.68	1.62 ± 0.68	0.136	−0.08 (−0.37, 0.21)	0.597
Legs	8.32 ± 2.41	8.14 ± 2.39	0.339	5.53 ± 2.33	5.94 ± 3.57	0.473	−0.59 (−1.72, 0.53)	0.303
Appendicular	11.06 ± 2.81	10.85 ± 2.89	0.472	7.10 ± 2.84	7.56 ± 4.09	0.423	−0.67 (−1.87, 0.53)	0.273
Muscle mass(kg)								
Arms	5.05 ± 1.90	5.01 ± 1.51	0.805	3.76 ± 0.79	3.78 ± 0.86	0.837	−0.06 (−0.45, 0.32)	0.743
Legs	15.80 ± 3.26	15.83 ± 3.44	0.808	12.23 ± 1.99	11.97 ± 2.12	0.256	0.29 (−0.21, 0.79)	0.259
Appendicular	20.85 ± 4.91	20.84 ± 4.83	0.975	15.98 ± 2.70	15.75 ± 2.90	0.415	0.22 (−0.51, 0.96)	0.549
Fat adiposity (%)								
Android	50.94 ± 4.98	51.00 ± 4.41	0.921	37.88 ± 8.02	40.53 ± 10.60	0.073	−2.59 (−5.46, 0.29)	0.078
Gynoid	41.36 ± 6.98	39.76 ± 7.32	0.030	38.68 ± 7.98	38.07 ± 10.52	0.639	−0.99 (−3.73, 1.75)	0.478
Muscle (%)								
Arms	63.37 ± 10.62	64.22 ± 10.15	0.143	71.09 ± 9.96	70.39 ± 10.61	0.281	1.56 (−0.04, 3.15)	0.055
Legs	65.48 ± 7.29	65.94 ± 7.43	0.283	69.42 ± 9.08	68.21 ± 12.12	0.445	1.68 (−1.39, 4.75)	0.284
Appendicular	65.01 ± 7.86	65.46 ± 7.90	0.197	69.70 ± 8.81	68.56 ± 11.49	0.412	1.59 (−1.07, 4.24)	0.242
Android	49.06 ± 4.98	49.00 ± 4.41	0.921	62.12 ± 8.02	59.47 ± 10.60	0.073	2.59 (−0.29, 5.46)	0.078
Gynoid	58.64 ± 6.98	60.24 ± 7.32	0.030	61.32 ± 7.98	61.93 ± 10.52	0.639	0.99 (−1.75, 3.73)	0.478
Whole body	60.45 ± 5.98	60.71 ± 5.89	0.573	69.04 ± 9.35	69.23 ± 7.64	0.891	0.07 (−2.58, 2.73)	0.957
Android/gynoid fat ratio	1.25 ± 0.18	1.31 ± 0.16	0.065	1.00 ± 0.23	1.13 ± 0.31	0.001	−0.07 (−0.15, 0.01)	0.105
Body fat percentage (%)	39.52 ± 5.96	39.29 ± 5.89	0.600	30.30 ± 7.97	30.86 ± 7.63	0.381	−0.79 (−2.23, 0.65)	0.283
ASMI	7.43 ± 1.39	7.40 ± 1.35	0.733	6.11 ± 0.68	6.02 ± 0.67	0.359	0.05 (−0.21, 0.31)	0.706
Forced vital capacity (%)	74.93 ± 16.19	78.00 ± 10.32	0.418	70.95 ± 20.27	77.00 ± 19.85	0.288	−2.95 (−15.44, 9.55)	0.644
Walk speed (m/s)	1.06 ± 0.30	1.00 ± 0.25	0.130	1.14 ± 0.21	1.17 ± 0.29	0.507	−0.10 (−0.23, 0.03)	0.116
Right grip (kg)	29.93 ± 12.42	34.07 ± 14.77	0.012	26.35 ± 9.12	27.75 ± 9.71	0.424	2.74 (−1.48, 6.96)	0.203
Left grip (kg)	27.21 ± 12.44	32.21 ± 12.89	0.001	27.20 ± 9.81	27.70 ± 9.40	0.707	4.50 (1.17, 7.83)	0.008
Quality of life score (*n* = 32)	17.69 ± 13.09	12.46 ± 14.05	0.259	13.00 ± 9.79	10.68 ± 11.19	0.132	−2.99 (−11.26, 5.29)	0.480
QMG total score (*n* = 32)	11.85 ± 4.72	9.69 ± 4.85	0.063	9.53 ± 4.71	8.53 ± 5.53	0.205	−0.21 (−2.97, 2.55)	0.883

MG, myasthenia gravis; CI, confidence interval; QMGS, quantitative myasthenia gravis scores.

**Table 6:** Body composition in patients with MG before and after resistance training according to QMGS score.

Variable	QMG Score ≤ 10			QMG Score > 11			Mean Difference (95% CI)	*p* for Interaction
	Pre-Test (*n* = 14)	Post-Test (*n* = 14)	*p*	Pre-Test (*n* = 20)	Post-Test (*n* = 20)	*p*		
Body mass index (kg/m^2^)	24.94 ± 5.48	24.60 ± 5.27	0.266	24.70 ± 4.06	25.04 ± 4.00	0.100	0.68 (0.01, 1.35)	0.046
Fat mass (kg)								
Arms	1.86 ± 0.86	1.99 ± 0.93	0.296	2.18 ± 0.95	2.12 ± 0.86	0.339	−0.20 (−0.46, 0.06)	0.139
Legs	6.58 ± 3.33	6.26 ± 3.06	0.144	6.75 ± 2.27	7.27 ± 3.45	0.363	0.84 (−0.28, 1.96)	0.143
Appendicular	8.44 ± 3.96	8.25 ± 3.85	0.501	8.93 ± 3.07	9.38 ± 4.05	0.436	0.64 (−0.56, 1.84)	0.294
Muscle mass(kg)								
Arms	4.31 ± 1.85	4.47 ± 1.57	0.392	4.27 ± 1.22	4.15 ± 1.11	0.156	−0.28 (−0.65, 0.09)	0.138
Legs	14.21 ± 3.38	14.03 ± 3.63	0.381	13.33 ± 2.93	13.24 ± 3.12	0.638	0.09 (−0.45, 0.63)	0.736
Appendicular	18.53 ± 4.99	18.50 ± 5.09	0.930	17.61 ± 4.09	17.39 ± 4.16	0.389	−0.19 (−0.97, 0.59)	0.634
Fat adiposity (%)								
Android	41.64 ± 8.96	42.97 ± 8.43	0.135	44.39 ± 9.85	46.15 ± 10.96	0.219	0.43 (−2.64, 3.51)	0.782
Gynoid	38.25 ± 9.08	36.66 ± 9.59	0.050	40.86 ± 6.39	40.24 ± 8.95	0.631	0.98 (−1.79, 3.76)	0.488
Muscle (%)								
Arms	69.28 ± 11.17	69.36 ± 10.26	0.919	66.95 ± 10.70	66.79 ± 11.16	0.762	−0.25 (−2.08, 1.58)	0.789
Legs	69.23 ± 10.33	69.80 ± 9.13	0.441	66.79 ± 7.07	65.51 ± 11.04	0.400	−1.86 (−5.03, 1.31)	0.249
Appendicular	69.27 ± 10.26	69.65 ± 9.22	0.586	66.72 ± 7.38	65.63 ± 10.67	0.411	−1.46 (−4.24, 1.32)	0.302
Android	58.36 ± 8.96	57.03 ± 8.43	0.135	55.61 ± 9.85	53.85 ± 10.96	0.219	−0.43 (−3.51, 2.64)	0.782
Gynoid	61.75 ± 9.08	63.34 ± 9.59	0.050	59.14 ± 6.39	59.76 ± 8.95	0.631	−0.98 (−3.76, 1.79)	0.488
Whole body	66.12 ± 10.17	67.67 ± 8.07	0.384	65.08 ± 8.52	64.35 ± 8.01	0.191	−2.28 (−5.70, 1.14)	0.191
Android/gynoid fat ratio	1.12 ± 0.21	1.22 ± 0.26	<0.001	1.10 ± 0.27	1.19 ± 0.28	0.018	−0.002 (−0.08, 0.08)	0.959
Body fat percentage (%)	32.59 ± 9.06	32.37 ± 8.06	0.760	35.16 ± 8.10	35.70 ± 7.97	0.293	0.75 (−0.86, 2.37)	0.359
ASMI	6.71 ± 1.39	6.65 ± 1.44	0.653	6.62 ± 1.10	6.55 ± 1.04	0.335	−0.005 (−0.29, 0.28)	0.973
Forced vital capacity (%)	74.29 ± 13.50	77.64 ± 12.42	0.241	71.42 ± 21.72	77.26 ± 18.97	0.329	2.44 (−9.67, 14.56)	0.692
Walk speed (m/s)	1.14 ± 0.12	1.24 ± 0.24	0.101	1.08 ± 0.30	1.01 ± 0.27	0.065	−0.18 (−0.32, −0.05)	0.007
Right grip (kg)	33.93 ± 9.48	35.86 ± 13.11	0.217	23.55 ± 9.28	26.50 ± 10.26	0.103	1.02 (−3.31, 5.35)	0.644
Left grip (kg)	34.29 ± 10.58	34.71 ± 10.82	0.699	22.25 ± 7.93	25.95 ± 9.88	0.017	3.27 (−0.12, 6.67)	0.059
Quality of life score (*n* = 32)	10.79 ± 8.75	6.07 ± 8.22	0.068	18.11 ± 12.20	15.56 ± 13.41	0.408	1.16 (−5.80, 8.12)	0.743
QMG total score (*n* = 32)	6.07 ± 2.27	6.00 ± 3.94	0.935	13.89 ± 3.07	11.33 ± 4.96	0.005	−1.98 (−4.38, 0.43)	0.107

MG, myasthenia gravis; CI, confidence interval; QMGS, quantitative myasthenia gravis scores.

## Data Availability

All data supporting our conclusions are contained within the article.
